# Multi-Camera Based Setup for Geometrical Measurement of Free-Falling Molten Glass Gob

**DOI:** 10.3390/s21041041

**Published:** 2021-02-03

**Authors:** Mazhar Hussain, Mattias O’Nils, Jan Lundgren

**Affiliations:** STC Research Centre, Mid-Sweden University, Holmgatan 10, 85170 Sundsvall, Sweden; mattias.onils@miun.se (M.O.); jan.lundgren@miun.se (J.L.)

**Keywords:** image processing, glass, multi-camera, threshold methods

## Abstract

High temperatures complicate the direct measurements needed for continuous characterization of the properties of molten materials such as glass. However, the assumption that geometrical changes when the molten material is in free-fall can be correlated with material characteristics such as viscosity opens the door to a highly accurate contactless method characterizing small dynamic changes. This paper proposes multi-camera setup to achieve accuracy close to the segmentation error associated with the resolution of the images. The experimental setup presented shows that the geometrical parameters can be characterized dynamically through the whole free-fall process at a frame rate of 600 frames per second. The results achieved show the proposed multi-camera setup is suitable for estimating the length of free-falling molten objects.

## 1. Introduction

Characterizing molten material in industry without compromising its quality is a complex task. The high temperature environment is too harsh for direct measurements and so producers have to rely on indirect laboratory methods, with the associated risk of introducing contamination to the samples.

For molten materials such as glass, it is of the utmost importance that the characteristics remain consistent during the production of the final product. Characteristics such as viscosity, volume and weight [1] needed to be continuously monitored. The factors effecting the characteristics might be inside mechanical components of glass-forming machines, synchronization between different parts of the machine or any change in the raw material for example viscosity of glass melt [2].

Currently most of the monitoring is manual and is based on previous experiences, operator knowledge, and trial and error [3]. However, manual monitoring is slow, not continuous and not sufficiently accurate. The result is lost in production and too high rejection rate of the final product due to failure to meet quality requirements [2]. Therefore, continuous monitoring of the gob is required to ensure the quality of the glass products such as glass containers.

This paper proposes the use of a multi-camera setup to dynamically characterize small changes in the geometrical properties of free-falling molten material that are correlated to material characteristics [4]. The setup is demonstrated in a lab environment using a solid cylindrical object. Images of the free-falling object are captured by the high-speed multi-camera setup and processed with image-processing techniques. We evaluate the effectiveness of this technique as well as how the use of two cameras helps to improve the end-result, which is an accurate measurement of the length of the cylindrical object.

Shape analysis that includes the length, width and area of the solid cylindrical object in each falling position shows that the setup is able to accurately measure the length of free-falling molten materials such as glass gob.

## 2. Related Work

Different fields have adopted different approaches to measuring the size of objects of interest. In [5], information about the position of a camera, its height and pitch angle with respect to ground, and a vanishing point are used to determine the height of a person. However, the technique proposed in [5,6] are not suitable for the experiment presented in this paper, in which it is not possible to determine the vanishing point from the scene. Paper [7] proposes using a measurement method based on an edge detection algorithm to automatically segment images of oysters and thus determine the height and width of each oyster. The researchers use an object of known size to calculate the pixel size ratio that can be used to estimate the measurements, but the focus of the paper is mostly on the segmentation technique. Frantisek et al. [8] proposed a method to estimate human height from a single calibrated image. They estimated the angles from the scene to determine the height. The authors claim a 1% to 2% error with a reference height of 1700 mm in the real environment. However, when estimating human height, accuracy in millimetres is not a serious concern and this method might not be suitable for measuring smaller objects with high accuracy. The method used in paper [4] has similar approach but more simplified as the object of interest is spherical and possess no influence on measurements due to tilt or perspective errors. However, in this article the shape of the object and presented correction methods minimize the error induced due to tilt and perspective errors.

Another approach [9] uses triangulation and depth of field to estimate a person’s height. It is assumed that the camera is facing downwards and looking at the object from an angle. This approach is not suitable for our application as the end-point required to their equation will be inaccurate due to the cylindrical shape of our objects.

A system is needed that can measure free-falling cylindrical or oval objects with high accuracy and with no reference object in the scene. In this article, we present a suitable approach to achieve this goal.

## 3. Materials and Methods

**Figure 1:**
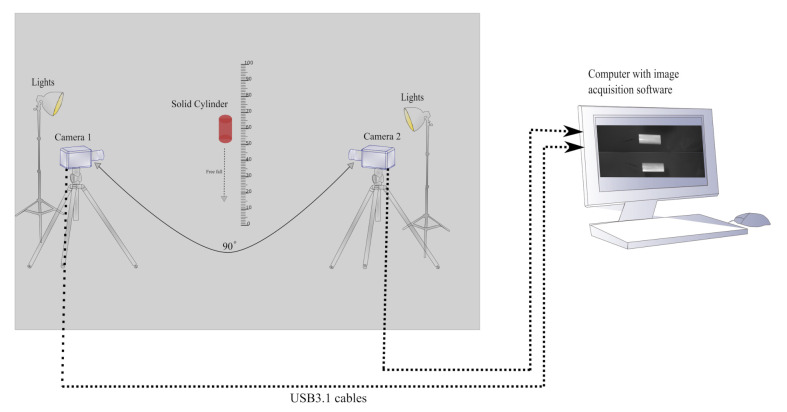
Measurement setup with two high-speed cameras placed 90 degrees apart and pointing to a free-falling solid cylinder. The object in red is a cylindrical object free-falling in front of the cameras.

The measurement setup consist of two high-speed cameras. The cameras are placed 90 degrees apart, covering the region of interest from different angles as shown in Figure 1. The geometrical parameters of the free-falling object are characterized continuously over the whole field of view (FOV) from the captured images.

The objective is to measure the length of the free-falling cylindrical object. Both cameras were first calibrated using a calibration pattern explained in [10]. Then the images of the cylindrical object were first converted into a binary image using the Otsu threshold method [11]. After detecting the boundaries, the images were cropped to remove background. The extracted object in the form of a bitmask was then used to determine the length of the object by first calculating the maximum Feret’s diameter [12] of the cylindrical object. Feret’s diameter is a caliper diameter that allows an object to be measured by placing it between the jaws of a Vernier caliper. Although simpler techniques to measure the length of the object could be used, the maximum Feret’s diameter was chosen for this experiment to determine whether this technique can accurately measure length in the intended application. A molten glass gob, which is a highly viscous liquid, may be subject to elongation during free-fall. We believe that the technique presented in this paper would represent the true length of the glass gob.

In this setup, camera 1 measures the length, and camera 2 provides a reference image to remove any tilt error in the measurements.

### 3.1. Proposed Approach to Measure the Length from One Camera

*Step 1*: Capture grayscale images of a free-falling cylinder as input from camera 1 and camera 2 simultaneously.

*Step 2*: Convert the grayscale images to binary images using the Otsu threshold method [11].

*Step 3*: Find the centroid $\bar{x},\bar{y}$, orientation θ, and major axis length *l* of the object in the binary image using moment-based geometrical properties [13].

*Step 3(a)*: The centroid $\bar{x},\bar{y}$ of the image is found using (1) and (2) [13].

(1) $$\bar{x}=\frac{1}{\left|R\right|}.\sum_{(u,v)\epsilon R}u$$

(2) $$\bar{y}=\frac{1}{\left|R\right|}.\sum_{(u,v)\epsilon R}v$$

where *R* is the binary region of the image, and *u, v* are the pixel co-ordinates.

We can define the binary image *(p,q)* moment of a contour *m* as:

(3) $$m_{p,q}=\frac{1}{\left|R\right|}.\sum_{(u,v)\epsilon R}I\left(u,v\right)u^{p}u^{q}$$

where *p* is the x-order and q is the y-order for I(u,v)ϵR.

For the binary image (u,v)ϵ{0,1}, only the foreground pixels with I(u,v)=1 in the region *R* are considered, giving a simplified form of (3):

(4) $$m_{p,q}=\frac{1}{\left|R\right|}.\sum_{(u,v)\epsilon R}u^{p}u^{q}$$

Now the area of the region *A(R)* can be expressed as:

(5) $$A\left(R\right)=\left|R\right|=\sum_{(u,v)\epsilon R}1=\sum_{(u,v)\epsilon R}^{n}u^{0}u^{0}\;=m_{00}\left(R\right)$$

(6) $$Centroid\left\{\bar{x},\bar{y}\right\}=\left\{\frac{m_{10}\left(R\right)}{m_{00}\left(R\right)},\frac{m_{01}\left(R\right)}{m_{00}\left(R\right)}\right\}$$

*Step 3(b)*: The major axis length *l* shown in Figure 1 has the same normalized second central moment of the region, that is, the central moment μ is the same as (4). The only difference is that now the values of *u* and *v* in the (7) are subtracted or displaced by the calculated centroid $\bar{x},\bar{y}$ in (1) and (2), which gives.

(7) $$\mu_{p,q}\left(R\right)=\sum_{(u,v)\epsilon R}I\left(u,v\right){\left(u-\bar{x}\right)}^{p}{\left(v-\bar{y}\right)}^{q}$$

For the binary image with I(u,v)=1 within region *R*, (7) becomes:

(8) $$\mu_{p,q}\left(R\right)=\sum_{(u,v)\epsilon R}{\left(u-\bar{x}\right)}^{p}{\left(v-\bar{y}\right)}^{q}$$

The normalized central moment is obtained by scaling the reciprocal of the area $m_{00}\left(R\right)$ as in (9) [14]:

(9) $${\bar{\mu }}_{p,q}\left(R\right)=\frac{\mu_{p,q}\left(R\right)}{\mu_{00}^{\frac{p+q}{2}+1}\left(R\right)}$$

where $\mu_{00}=m_{00}\left(R\right)$ and the central moments of order up to two are [15]

(10) $$\begin{array}{c} \mu_{00}=0,\;\\ \mu_{10}=0,\\ \mu_{11}=m_{11}\left(R\right)-\bar{x}*m_{01}\left(R\right)=m_{11}\left(R\right)-\bar{y}*m_{10}\left(R\right),\\ \mu_{20}=m_{20}\left(R\right)-\bar{x}*m_{10}\left(R\right),\\ \mu_{02}=m_{02}\left(R\right)-\bar{y}*m_{01}\left(R\right),\\ \mu_{21}=m_{21}\left(R\right)-2\bar{x}*m_{11}\left(R\right)-\bar{y}*m_{20}\left(R\right)+2x^{-2}*m_{01}\left(R\right),\\ \mu_{12}=m_{12}\left(R\right)-2\bar{y}*m_{11}\left(R\right)-\bar{y}*m_{02}\left(R\right)+2y^{-2}*m_{10}\left(R\right). \end{array}$$

The covariance matrix in (14) is constructed using the information from (10):

(11) $${\bar{\mu }}_{20}=\frac{\mu_{20}}{\mu_{00}}=\frac{m_{20}\left(R\right)}{m_{00}\left(R\right)-{\bar{x}}^{2}}$$

(12) $${\bar{\mu }}_{02}=\frac{\mu_{02}}{\mu_{00}}=\frac{m_{02}\left(R\right)}{m_{00}\left(R\right)-{\bar{y}}^{2}}$$

(13) $${\bar{\mu }}_{11}=\frac{\mu_{11}}{\mu_{00}}=\frac{m_{11}\left(R\right)}{m_{00}\left(R\right)-\bar{x}\bar{y}}$$

(14) $$\;\left[\begin{array}{cc} {\bar{\mu }}_{20} & {\bar{\mu }}_{11}\\ {\bar{\mu }}_{11} & {\bar{\mu }}_{02} \end{array}\right]$$

Equation (15) represents the major axis length *l* is approximated by constructing a covariance matrix of the normalized second central moment of the region *R* in (14). The eigenvectors of this matrix corresponds to the major $\lambda_{1}$ and minor axes $\lambda_{2}$ [16], calculated as:

(15) $$\lambda_{1,2}=\frac{{\bar{\mu }}_{20}+{\bar{\mu }}_{02}\pm \sqrt{{\left({\bar{\mu }}_{20}-{\bar{\mu }}_{02}\right)}^{2}-4{\bar{\mu }}_{11}}}{2{\bar{\mu }}_{00}}$$

where $\lambda_{1}$ takes the + sign and $\lambda_{2}$ takes the – sign, so that $\lambda_{1}\geq \lambda_{2}$.

(16) $$l=2\sqrt{\lambda_{1}}$$

*Step 3(c)*: Orientation θ is calculated by fitting an ellipse [13] over the whole object after extracting the major axis length*l*.

Using Pythagoras we can obtain (17) and (18):

(17) $$sin\left(2\theta_{R}\right)=\frac{A}{\sqrt{A^{2}+B^{2}}}$$

(18) $$cos\left(2\theta_{R}\right)=\frac{A}{\sqrt{A^{2}+B^{2}}}$$

where $A=2\mu_{11}\left(R\right)$ and $B=\mu_{20}\left(R\right)-\mu_{02}\left(R\right)$.

Now for region *R*, the orientation vector $xd={\left(x_{d},y_{d}\right)}^{T}$ is computed as in [12] using $cos^{2}\alpha =\frac{1}{2}\left[1+cos2\alpha \right]$ and $sin^{2}\alpha =\frac{1}{2}\left[1-cos2\alpha \right]$.

(19) $$x_{d}=cos\left(\theta_{R}\right)=\left\{\begin{array}{ll} 0 & \mathrm{for}A=B=0\\ {\left[\frac{1}{2}\left(1+\frac{B}{\sqrt{A^{2}+B^{2}}}\right)\right]}^{\frac{1}{2}} & \mathrm{otherwise} \end{array}\right.$$

(20) $$x_{d}=sin\left(\theta_{R}\right)=\left\{\begin{array}{ll} 0 & \mathrm{for}\;A=B=0\\ {\left[\frac{1}{2}\left(1-\frac{B}{\sqrt{A^{2}+B^{2}}}\right)\right]}^{\frac{1}{2}} & \mathrm{for}A\geq 0\\ {\left[\frac{1}{2}\left(1-\frac{b}{\sqrt{A^{2}+B^{2}}}\right)\right]}^{\frac{1}{2}} & \mathrm{for}A<0 \end{array}\right.$$

The orientation $\theta_{R}$ for the region *R* is calculated as:

(21) $$\theta_{R}=\frac{arctan(A,B)}{2}$$

**Figure 2:**
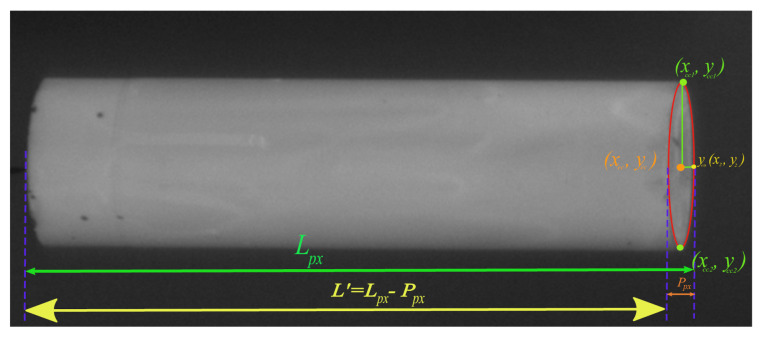
Ellipse fitted over the elliptical part of the cylindrical object along with measured length with and without the perspective error.

*Step 4*: To calculate the length of the cylindrical object, we use the major axis length *l* from (16) and orientation $\theta_{R}$ from (21) of the cylinder. We first extract the *x* and *y* co-ordinates of a line segment with high intensity values that starts from one end of the boundary to the other end of the region’s boundary and passes through the centroid $\left(\bar{x},\bar{y}\right)$ of the cylinder as shown in the Figure 2.

(22) $$k=\frac{1}{2}$$

(23) $$x_{co}=\bar{x}+k*\left[\begin{array}{cc} cos\theta_{R} & -cos\theta_{R} \end{array}\right]$$

(24) $$y_{co}=\bar{x}+k*\left[\begin{array}{cc} -sin\theta_{R} & sin\theta_{R} \end{array}\right]$$

*Step 5*: From Figure 2 first find the last index of the points with high intensity values for both $x_{c0}=\left(x_{1},y_{1}\right)$ and $y_{c0}=\left(x_{2},y_{2}\right)$ co-ordinates.

*Step 6*: Calculate the Euclidean distance for the length in pixels $L_{px}$ as shown in (25).

(25) $$L_{px}=\sqrt[{2}]{{\left(x_{1}-x_{2}\right)}^{2}+{\left(y_{1}-y_{2}\right)}^{2}}$$

*Step 7*: Divide the length in pixels by the camera’s pixel size ratio $\left(\frac{px}{mm}\right)$ to get the length in millimetres, *L*, as in (26).

(26) $$L=\frac{L_{px}}{\left(\frac{px}{mm}\right)}$$

**Figure 3:**
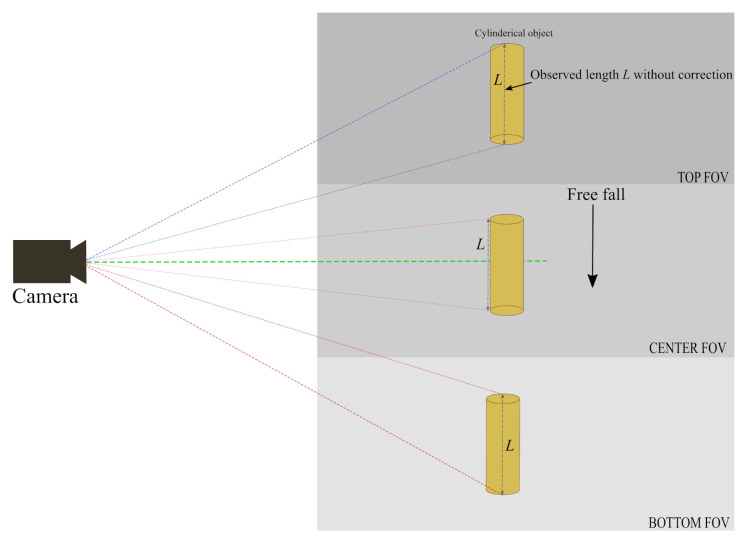
Observed length of cylindrical object from top, centre and bottom of vertical Field of View (VFOV) of the camera.

Perspective errors can occur at the top or bottom elliptical parts of the cylindrical object (the ends) due to camera angle, as shown in Figure 3. The minor axis of this elliptical part is added to the binary image and becomes part of the measured length, adding perspective error to the measurements. To eliminate this error, the centre of this elliptical part is calculated. Minor and major axes are then extracted to fit an ellipse as shown in Figure 2. The minor axis can then be subtracted from the measured length. The length of the cylindrical object before and after correction is shown in the results section of this paper.

**Figure 4:**
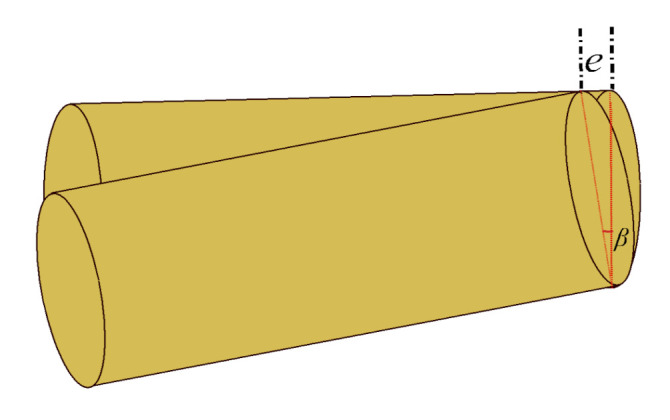
Object with tilt error *e*.

*Step 8*: The diameter of the minor axis of the elliptical part $P_{px}$ shown in Figure 4 can be calculated using (27):

(27) $$P_{px}=\left(\sqrt[{2}]{{\left(x_{2}-x_{ec}\right)}^{2}+{\left(y_{2}-y_{ec}\right)}^{2}}\right)*2$$

*Step 9*: The perspective correction in length *L* shown in Figure 4 of the cylindrical object can now be achieved by subtracting $P_{px}$ from $L_{px}$. Equation (28) shows the correct length in pixels $L_{px}^{\prime }$.

(28) $$L_{px}^{\prime }=L_{px}-P_{px}$$

Equation (28), which is in pixels, is divided by the camera’s pixel size ratio $\left(\frac{px}{mm}\right)$ to obtain the length in millimetres.

(29) $$L^{\prime }=\frac{L_{px}^{\prime }}{\left(\frac{px}{mm}\right)}$$

### 3.2. Proposed Method to Remove Tilt Error Using Two Cameras

Using the images from the two cameras pointing at the same object from two different viewpoints, 90 degrees apart as shown in Figure 1, any tilt in a falling object can be identified and rectified in post-processing. Figure 4 shows the method of calculating the tilt error *e* and then eliminating that error from the length $L_{px}^{\prime }$.

*Step 10*: The tilt error is calculated with the orientation angle β as shown in Figure 4. The orientation angle β is taken from the reference camera (i.e., camera 2).

Equation (30) gives the tilt error *e*:

(30) e=rtanβ

*Step 11*: To measure the width of the object, the minimum Feret’s diameter of the object is determined from the binary image.

*Step 12*: Repeat *step 2* to *step 3* on the images taken from camera 2.

**Figure 5:**
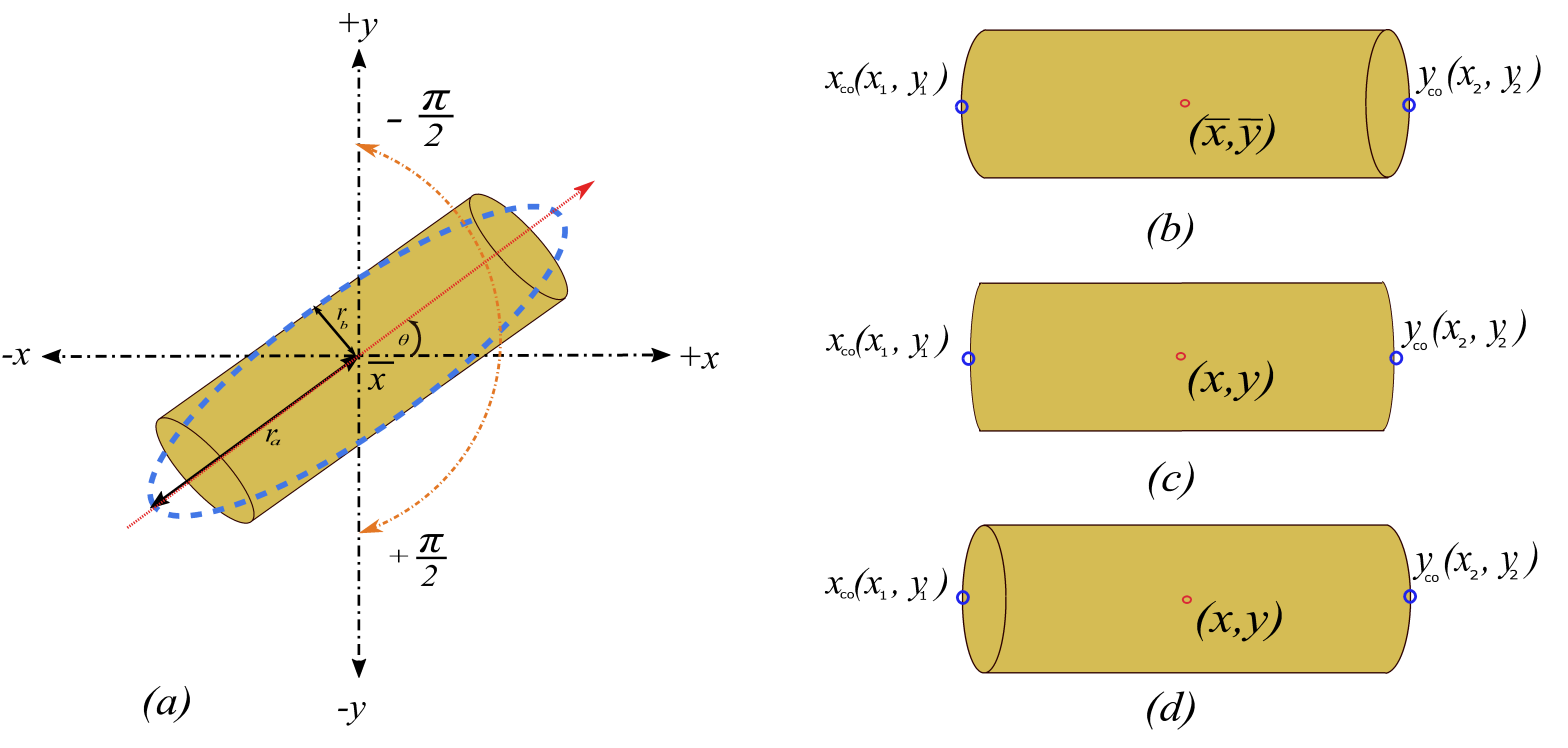
(**a**) Region orientation, with the major axis of the region passing through the centroid ( $\bar{x},\bar{y}$) with orientation θ [13]. The *x* and *y* are the co-ordinates of a line segment of the binary image with high intensity values. Perspective errors can also be seen when the object is viewed from the (**b**) top FOV (**c**) centre FOV, and (**d**) bottom FOV.

*Step 13*: From a binary image with labelled matrix, the minimum Feret’s diameter *r* is measured between two boundary points on the antipodal vertices of a convex hull that encloses the cylindrical object. $r_{b}$ in Figure 5a shows the distance from the centroid to one of the boundary points on the antipodal vertices and can be calculated as:

(31) $$r_{b}=2\sqrt{\lambda_{2}}$$

where $\lambda_{2}$ is taken from (15).

The width of the cylinder *r* is calculated from $r_{b}$ from (31):

(32) $$r=2*r_{b}$$

*Step 14*: After calculating the tilt error *e* from the images taken from camera 2, the tilt error *e* is added into the length in pixels $L_{px}^{\prime }$ from (28).

(33) $$L_{px}^{\prime \prime }=L_{px}^{\prime }-e$$

*Step 15*: Convert the length $L^{\prime \prime }$ to millimetres by dividing the length in pixels $L_{px}^{\prime \prime }$ by the camera’s pixel size ratio $\left(\frac{px}{mm}\right)$ as shown in (34):

(34) $$L^{\prime \prime }=\frac{L_{px}^{\prime \prime }}{\left(\frac{px}{mm}\right)}$$

## 4. Results

An experiment was designed to evaluate the proposed multi-camera setup and measurement methods described above. The experimental setup for this paper consists of two high-speed uEye UI-3060CP-M-GL-R2 monochrome cameras with 25 mm fixed focal length lenses. The selected frame rate for this experiment was 600 fps, with a cropped region of interest of 1936 × 300 pixels. This gives a combination of relatively large number of pixels in the free-fall direction for geometrical characterization with low quantization error. This enables high resolution of the free-fall as the expected velocity of the falling cylinder is around 4 m/s; for this frame rate, the cylinder will only move 5.7 mm in each frame [17]. The exposure time was selected on the basis of the expected motion blur. For this experiment the calculated exposure time was 0.132 ms. The motion blur due to the chosen exposure time was calculated as stated in [17]. The motion blur will be 0.528 mm, which is adequate considering the size of the cylindrical object used in this experiment. Both cameras were placed 90 degrees apart with a working distance of 200 cm, facing towards the solid cylindrical object with true length of 186 mm (Figure 1).

**Figure 6:**
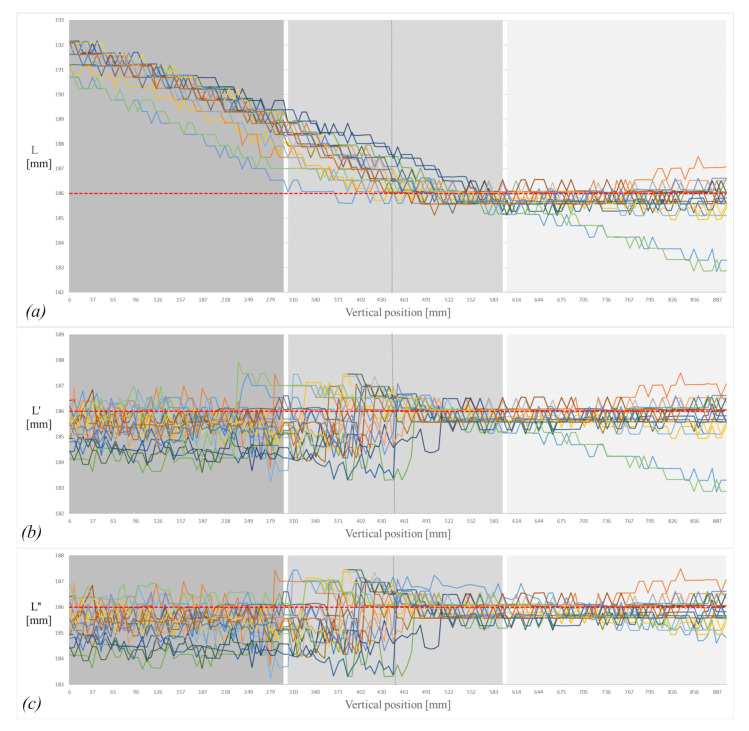
(**a**) Measured length without correction; (**b**) measured length with perspective correction; (**c**) measured length with tilt correction. Note that different colors represent each drop with length measurements of the falling cylinder.

**Figure 7:**
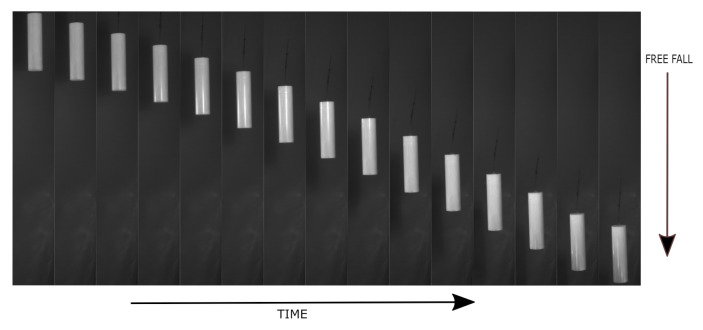
Sample images of free-falling object from setup.

The object was dropped from 100 cm above the ground. Both cameras covered the vertical field of view (VFOV), that is, 90 cm in such a way that the pixel size ratio would be 2.15 pixels/mm for camera 1 and 2.14 pixels/mm for camera 2. The pixel size ratio was calculated by dividing the resolution of the camera 1936 *px* by the VFOV. The object was dropped 20 times. In Figure 6a–c, different colors represent each drop with length measurement of the falling cylinder. Figure 7 shows a sequence of images for the free-falling solid cylindrical object captured in the experimental setup.

**Figure 8:**
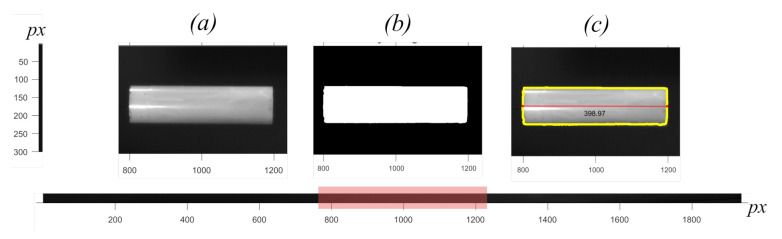
Measured length from image of falling cylindrical object taken from setup. (**a**) original image, (**b**) binary image, (**c**) original image with boundaries and length $\left(L_{px}\right)$.

Figure 8 shows the processing steps from captured image to binary image to measured length in pixels $L_{px}$.

The different methods for measuring the object length described in the method section were applied to the captured images. Measurement of raw object length based on (25) is shown in Figure 6a. The measured object length with perspective compensation, (29) is presented in Figure 6b. The two camera-based tilt compensation (34) is shown in Figure 6c.

### Length Estimation of a Falling Glass Gob

**Figure 9:**
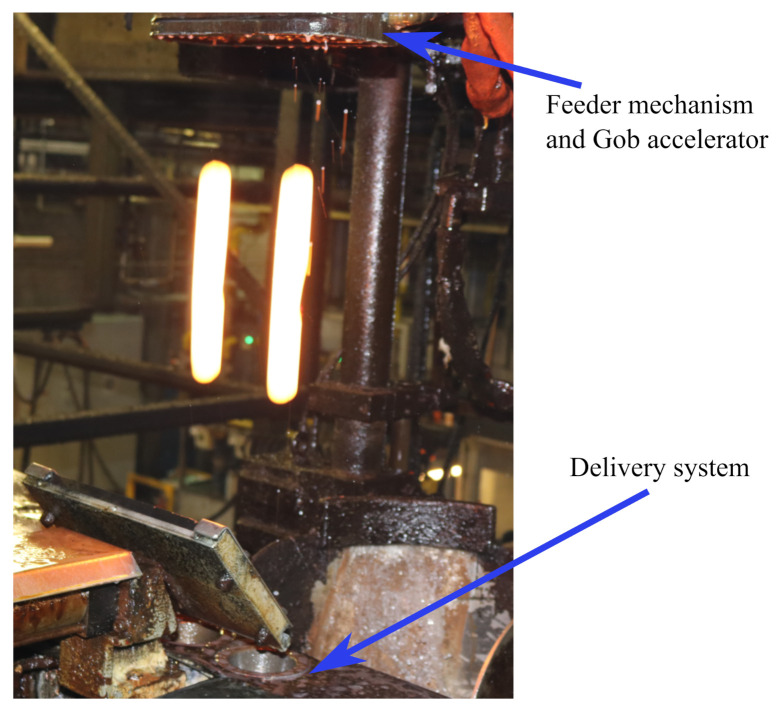
Free-falling glass gobs between Feeder mechanism and delivery system.

**Figure 10:**
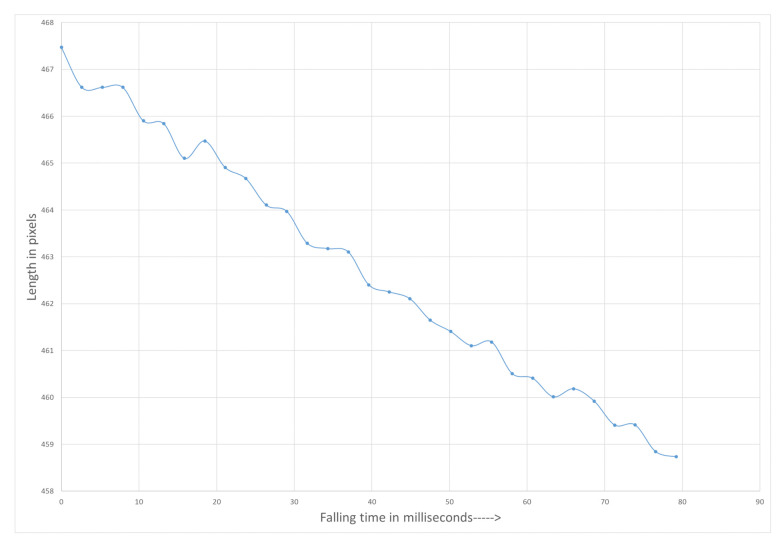
Measured length of free falling glass gob.

Measurement setup was installed on a real glass plant between the Feeder mechanism and Delivery system. The addition to the measurement setup a high speed thermal camera was placed to get the temperature of the glass gob. The recorded temperature range was between 1090 °C to 1125 °C. The VFOV was limited to 75.6 cm due to the presence of Gob accelerator below the Feeder mechanism. The working distance was 170 cm. Figure 9 shows the two falling gobs (after being cut by shear mechanism located inside Feeder) between the Feeder and Delivery system. Length of the falling gob was measured and corrected with proposed method. Figure 10 shows the length (in pixels) of gob at different falling positions.

## 5. Discussion

This paper has proposed and evaluated a multi-camera setup for geometrical characterization of free-falling objects. The setup was designed to evaluate its ability to measure the length of an object accurately and dynamically with high temporal resolution during free-fall as illustrated in Figure 7. The intended application is to measure a free-falling molten glass gob. The drop height in this setup was selected based on the average spacing between the fore-hearth (feeder) and the delivery system of the actual glass container machine [18].

**Figure 11:**
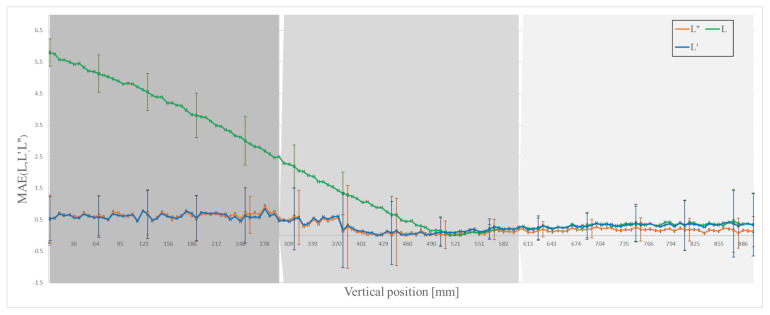
Mean absolute error (MAE) with standard deviation in measured and corrected length.

The captured image is processed and analysed to measure the length of the object. The processed image is also used to estimate the perspective error caused by the elliptical part of the object. Figure 11 shows the effectiveness of this technique. The perspective error is reduced to 0.43 for *L’* with standard deviation of 0.69 mm for full FOV and relative accuracy of 99.8 %. Considerable reduction in perspective error can be seen near the top and the centre part of the FOV where the error is reduced from 4.32 to 0.62 with a standard deviation of 0.74, and 1.07 to 0.28 mm with a standard deviation of 0.53 mm, respectively (see Table 1 and Table 2).

Camera 2, at an angle of 90 degrees apart from camera 1 and also focused on the free-falling object, is used to identify the error caused by tilt in the falling object. Tilt towards or away from the camera makes an object appear smaller than its actual length. If the object is tilted that is towards or away from camera 1, the object will appear to be tilted sideways to camera 2. Figure 6c shows the correction in length due to this tilt error. The results in Table 1 and Figure 11 show that the total error due to both perspective and tilt is reduced to 0.41 with a standard deviation of 0.6 mm and relative accuracy of 99.8 % for full FOV. The largest reduction in error is in the centre and the bottom part of the FOV: the error is reduced to 0.25 with standard deviation of 0.53 mm, and 0.18 with standard deviation of 0.36 mm, respectively (see Table 1 and Table 2).

In Figure 6a–c slight deviations and variability in measurement are due to segmentation error, which is about 0.16%. That means the standard deviation in *L”* (Table 1) is basically due to segmentation error.

The segmentation error may increase where there is insufficient illumination. The error can be reduced by using better illumination and by setting the binary threshold manually. However, this is not an issue in the intended application as a molten glass gob glow due to its very high temperature.

**Figure 12:**
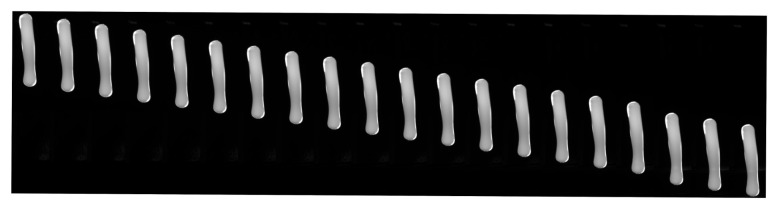
Images of free-falling glass gob from setup.

Figure 9 shows the actual free fall of a glass gob between feeder and delivery system. The proposed method to measure length is applied and presented in Figure 10 and Figure 12. The gob deforms and started to shrink during the free fall.

Results shows that proposed simple method gives good accuracy in measurement, which gives small opportunities for improved accuracy with more advanced setups for example stereo vision. However, due to harsh environment careful configuration of the setup is still required when the setup is placed in real plant. An addition of a stereo vision to automatically calculate all the setup parameter can remove the need of careful configuration of the setup.

In glass container production, different sizes of containers produced which eventually require various size of gobs. Molten gobs can be cut in small chunks or big depending on the size of the required glass container. The curvature of the gob on top and bottom part may change with the size of glass gob. Further investigation and comparison between the shapes of gobs is required.

## 6. Conclusions

This paper shows that the size of a cylindrical object can be characterized in free-fall with 99.8% accuracy with either one camera or two cameras. Using two cameras makes the setup more robust against object tilt. The relative accuracy with one camera might decrease and the RMSE might increase as the number of tilted cylinders increases. The results show that a higher accuracy can be achieved if the observation area is limited to the centre/bottom part of the camera FOV. The results presented show that the proposed camera-based setup can characterize dynamic geometrical changes in a free-falling object such as a molten glass gobs, enabling contactless analysis of the characteristics of the glass. Further, the results show that if the falling object has limited tilt, one camera will give as good results as two will, but two cameras will remove errors caused by tilted objects. The proposed method of length measurement is applied on the free falling glass gob and results shows that there are geometrical changes on the free falling glass gobs that require further investigation to correlate the results with glass characteristics. During the measurements on glass plants, it was observed that careful configuration of the setup is required, and an addition of stereo vision could overcome this issue.

## Figures and Tables

**Table 1:** Root mean square error (RMSE) and standard deviation (SDE) for length estimation methods and selected field of view (FOV).

FOV	L		L’		L”	
	RMSE	SDE	RMSE	SDE	RMSE	SDE
Full	2.57	0.59	0.43	0.69	0.41	0.60
Top	4.32	0.61	0.62	0.74	0.64	0.70
Centre	1.07	0.53	0.28	0.53	0.25	0.53
Bottom	0.33	0.60	0.31	0.60	0.18	0.36

**Table 2:** Relative accuracy of the length measurements for the different length estimation methods and selected field of view (FOV) in percent (%).

FOV	L	L’	L”
Full	99	99.8	99.8
Top	97.8	99.7	99.7
Centre	99.6	99.9	99.9
Bottom	99.8	99.8	99.9

## Data Availability

Not applicable.
